# A peptide for targeted, systemic delivery of imaging and therapeutic compounds into acute brain injuries

**DOI:** 10.1038/ncomms11980

**Published:** 2016-06-28

**Authors:** Aman P. Mann, Pablo Scodeller, Sazid Hussain, Jinmyoung Joo, Ester Kwon, Gary B. Braun, Tarmo Mölder, Zhi-Gang She, Venkata Ramana Kotamraju, Barbara Ranscht, Stan Krajewski, Tambet Teesalu, Sangeeta Bhatia, Michael J. Sailor, Erkki Ruoslahti

**Affiliations:** 1 Cancer Research Center, Sanford Burnham Prebys Medical Discovery Institute, La Jolla, California 92037, USA; 2 Laboratory of Cancer Biology, Institute of Biomedicine and Translational Medicine, University of Tartu, 50411 Tartu, Estonia; 3 AivoCode, La Jolla, California 92037, USA; 4 Department of Chemistry and Biochemistry, University of California, San Diego, La Jolla, California 92093, USA; 5 Broad Institute of Harvard and MIT, Cambridge, Massachusetts 02142, USA; 6 Institute for Medical Engineering and Science, and Howard Hughes Medical Institute, Massachusetts Institute of Technology, Cambridge, Massachusetts 02139, USA; 7 Center for Nanomedicine and Department of Cell, Molecular and Developmental Biology, University of California, Santa Barbara, California 93106, USA

## Abstract

Traumatic brain injury (TBI) is a major health and socio-economic problem, but no pharmacological agent is currently approved for the treatment of acute TBI. Thus, there is a great need for advances in this field. Here, we describe a short peptide (sequence CAQK) identified by *in vivo* phage display screening in mice with acute brain injury. The CAQK peptide selectively binds to injured mouse and human brain, and systemically injected CAQK specifically homes to sites of brain injury in mouse models. The CAQK target is a proteoglycan complex upregulated in brain injuries. Coupling to CAQK increased injury site accumulation of systemically administered molecules ranging from a drug-sized molecule to nanoparticles. CAQK-coated nanoparticles containing silencing oligonucleotides provided the first evidence of gene silencing in injured brain parenchyma by systemically administered siRNA. These findings present an effective targeting strategy for the delivery of therapeutics in clinical management of acute brain injuries.

## Abstract (web summary)

Accurate treatment of traumatic brain injuries, a leading cause of neurological disability and death in young people, is hampered by poor accumulation of drugs in the brain. Here, the authors describe a tetrapeptide that can efficiently target brain injuries and deliver therapeutic or diagnostic payload.

## Graphical abstract

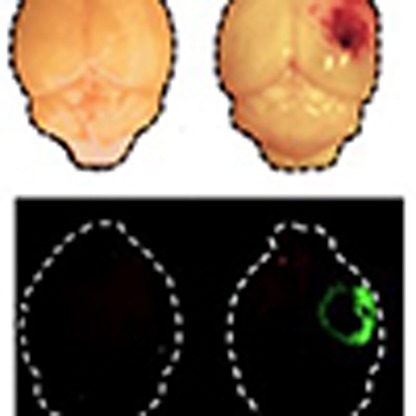

Acute brain injury such as traumatic brain injury (TBI) disrupts the normal function of the brain and generally has a poor prognosis for functional recovery and survival. Termed a ‘silent epidemic', TBI is a leading cause of mortality and morbidity in children, teens and active adults from ages 1 to 44, with an annual incidence of 2.5 million in the US (ref. 1). TBI can lead to acute and potentially long-lasting neurological dysfunction, including the development of chronic traumatic encephalopathy or even Alzheimer's disease^2^. A majority of combat-related TBI cases are additionally complicated by a penetrating injury to the brain, which is often even more difficult to manage than non-penetrating injuries^3^. Despite this substantial socio-economic impact, TBI treatment is limited to palliative care and no specific therapies with long-term benefits are available.

The blood–brain barrier (BBB) is considered a major impediment to systemic treatment of central nervous system (CNS) diseases. As a result, localized delivery of drugs within the brain has been explored, but it has limitations in clinical settings. In acute brain injury and several cerebrovascular diseases, including stroke, hypertension and ischaemia, the BBB is transiently disrupted, which allows extravascular access for macromolecules and neuroprotective drugs from the systemic circulation. In fact, the leakage of serum proteins into brain parenchyma is used to test for BBB integrity^4^. However, lack of specific binding of passively accumulating proteins in the injured area can result in low retention and subsequent washout over time. Due to this clearance, the therapeutic efficacy of a systemically administered drug may be greatly limited.

We previously employed *in vivo* phage display as a powerful and unbiased method to probe tissues *in situ* for specific molecular signatures and discovered homing peptides specific for different pathologies including tumours, atherosclerotic plaques and wounds^5^,^6^,^7^. We reasoned that an acute and complex event such as TBI is suited for a similar approach as site-specific molecular changes in protein expression have been reported^8^. In this study, we set out to identify peptides that would recognize specific molecular changes at the sites of traumatic injury in the brain, and enhance delivery of therapeutic compounds to such sites. The goal of this approach was to explore an alternative to local delivery of therapeutics, which is invasive and can add complications to the injury.

## Results

### Isolation of brain injury selective peptide by phage display

**Figure 1:**
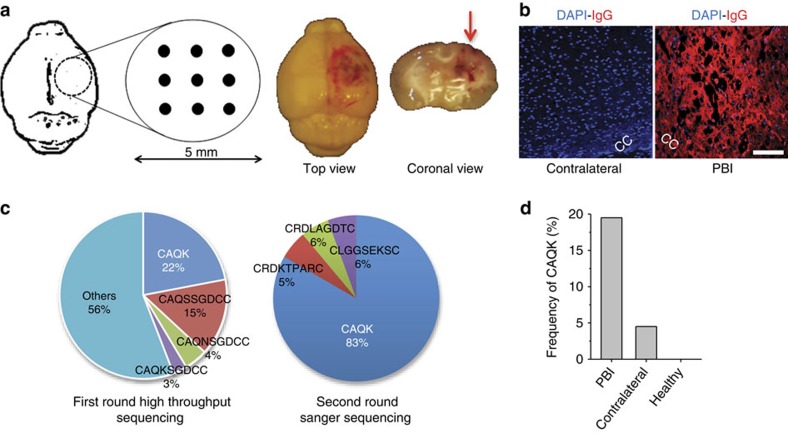
***In vivo*** **phage screening in PBI.** (**a**) Schematic of the PBI mouse model, wherein a 5-mm craniotomy was performed in the right parietotemporal cortex and nine needle punctures were inflicted according to the grid shown. Right panel shows perfused brain, 6 h after the unilateral injury, from the top and in coronal view. (**b**) Representative immunofluorescence images show leakage through compromised BBB in PBI. Perfused PBI brain at 6 h was stained in the region around the corpus callosum (cc) for mouse IgG (red) and 4,6-diamidino-2-phenylindole (DAPI; blue). Scale bar, 50 μm. (**c**) Summary of peptide sequences recovered from phage screening in PBI mice. CAQK phage preferentially accumulated in brain injury after systemic injection of naïve phage library (1 × 10^9^ p.f.u.) in PBI animals; rescued first round phage pool sequenced with high-throughput sequencing showed CAQK and its variants (left pie chart). Sequences from second round of biopanning (right pie chart) show high CAQK recovery confirmed through Sanger sequencing. (**d**) CAQK phage frequency in brain as per cent of total phage recovered. Compared with PBI, CAQK was present at a lower percentage in the contralateral hemisphere in injured mice and absent in healthy, control mice.

To isolate peptides that specifically target brain injury, we inflicted unilateral puncturing stab wound injuries to the right hemisphere of adult male mice (Fig. 1a). The penetrating brain injury (PBI) resulted in rupturing of BBB visualized by selective leakage of mouse IgG into the brain parenchyma on the injured side (Fig. 1b). PBI also caused cortical tissue loss, axonal damage and loss of myelin in the corpus callosum (Supplementary Fig. 1b), and was accompanied by an increase in glysocaminoglycan deposition in the injured hemisphere (Supplementary Fig. 1c).

A T7 phage library that displays on the phage surface 9-amino acid cyclic peptides with the general composition of CX7C (C=cysteine; X=any amino acid)^7^ was intravenously injected 6 h after PBI. Phage was harvested 30 min after injection from the injury site and the corresponding contralateral hemisphere. Phage recovery was 10-fold higher from the injured hemisphere than from the uninjured contralateral side, indicating BBB breakdown caused by the injury. High-throughput sequencing analysis of the recovered phage pool revealed a striking enrichment of phage with the tetra-peptide insert, CAQK, which comprised 22% (1.28 × 10^5^ p.f.u.) of total recovered phage pool (6.4 × 10^5^ p.f.u.; Fig. 1c). In addition to the truncated CAQK peptide, full-length (9aa) cyclic inserts starting with the CAQK motif were also recovered at lower frequency. A second round of biopanning increased the CAQK fraction to 83% of the total recovered phage pool. Interestingly, there was some CAQK phage recovery from the contralateral side (4%), which suggested a mild impairment triggered through the contralateral injury (Fig. 1d). No CAQK was recovered from the brain of a normal mouse injected with the phage library.

**Figure 2:**
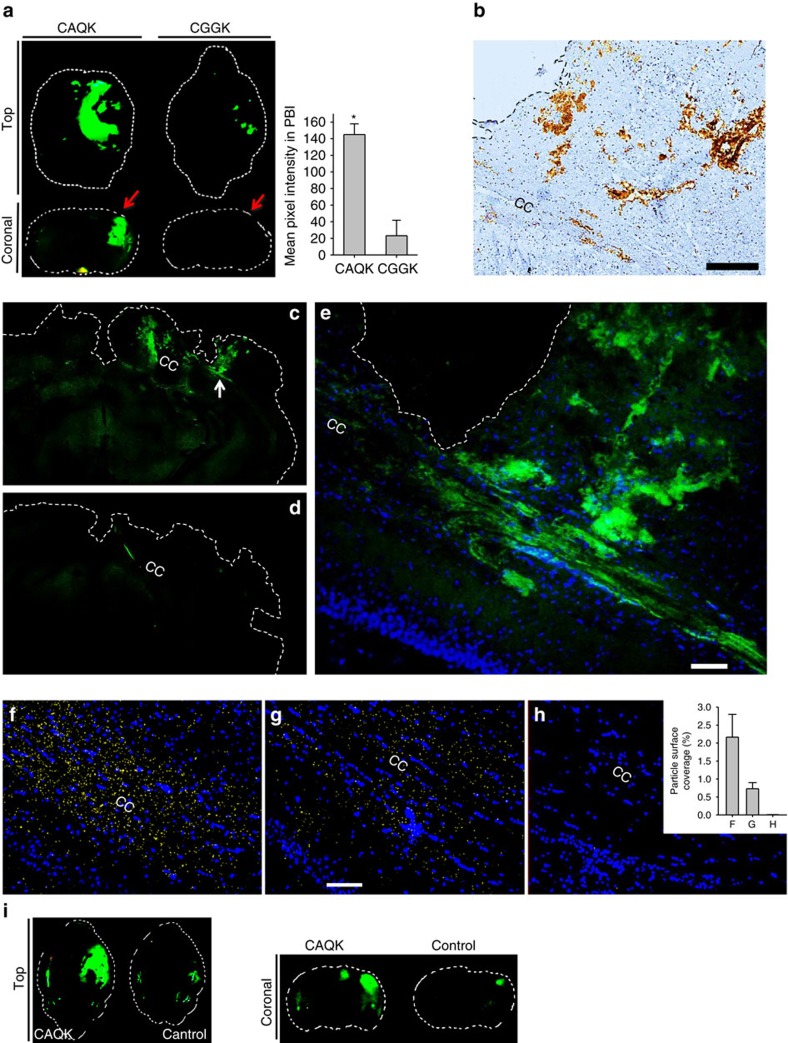
CAQK peptide shows selective homing to PBI. (**a**) Fluorescence brain images (top view and coronal view) of mice injected with FAM-labelled peptides 6 h after PBI. Animals were perfused, brains isolated and imaged under an Illumatool System (green channel). (*P<0.05, ANOVA analysis, *n*=6); mean±s.e.m. (**b**) FAM-CAQK in the sections from injured brain was visualized by immunohistochemical staining for FAM (brown) from FAM-CAQK in PBI. Sections counterstained with haematoxylin brains isolated (blue). Scale bar, 100 μm. (**c**–**e**) Fluorescence signal of FAM-CAQK (C) and FAM-Control (D) in clarified PBI brains. Peptides injected 6 h after PBI were allowed to circulate for 30 min followed by the CLARITY protocol for clearing brains. (**e**) shows higher magnification from **c** (white arrow points to the magnified region). CAQK showed homing to fibre-like structures in the corpus callosum (CC) area. Scale bar, 50 μm. (**f**–**h**) CAQK conjugated AgNPs bind to PBI sections around the CC. Overlay experiments using peptide-conjugated AgNPs on frozen PBI sections counterstained with 4,6-diamidino-2-phenylindole (DAPI; blue), green AgNPs were pseudo coloured to yellow for higher colour contrast, and the threshold was equally enhanced for all samples using Image J. Shown are representative sections of CAQK-NP binding to PBI sections (**f**), CAQK-NP binding to healthy brain sections (**g**) and control-NP binding to PBI sections (**h**). The number of particles in three fields were counted and plotted in the bar diagram from three brains. Scale bar, 50 μm. Mean±s.e.m. (**i**) Fluorescence brain images (top view and coronal view) of mice injected with FAM-labelled peptides 6 h after a controlled cortical injury.

To validate the selectivity of CAQK, a fluoresceinamine (FAM)-labelled CAQK peptide was chemically synthesized. In agreement with the phage screening results, the synthetic FAM-CAQK peptide, when injected intravenously, showed selective homing to the injured brain on macroscopic examination (Fig. 2a), and immunohistochemical staining for the FAM label on the peptide (Fig. 2b). A FAM-labelled control peptide (CGGK) of the same length and overall charge as CAQK yielded minimal fluorescent signal. No CAQK accumulation was observed in healthy brain (Supplementary Fig. 2a) or in other major tissues (Supplementary Fig. 2b) after 30 min circulation, except in the kidney, which is the common route for peptide elimination from the circulation.

To investigate if CAQK targets other types of brain injuries, we tested peptide homing in a controlled cortical impact injury (CCI) model. Without penetrating injury, this model mimics cortical tissue loss, axonal injury, concussion and BBB dysfunction of TBI (ref. 9). CAQK homed to the injured area in the brain in this model (Fig. 2i). Binding of CAQK peptide was specific to brain injuries as no peptide accumulation was detected in perforating injuries inflicted on the liver and skin (Supplementary Fig. 2c,d). These findings suggest that the binding epitope for CAQK peptide is specific for sites of brain injury, at least in the models tested.

CAQK homing to PBI was observed up to 5 days after the injury (Supplementary Fig. 3), indicating a potential window for effective systemic CAQK-targeting. To visualize peptide accumulation across the entire brain, whole brains were processed and made transparent using the CLARITY protocol^10^ and FAM-CAQK was visualized in the transparent tissue. FAM-CAQK signal in the clarified brain was restricted to the injured quadrant of the brain and was higher than the signal from FAM-CGGK control (Fig. 2c,d). CAQK accumulated in the region of the commissural fibres of the corpus callosum and in ring-shaped cellular structures in the cortex (Fig. 2e). CAQK accumulation led to a greater retention in the injured brain, as the peptide signal was visible 3 h after injection whereas the control peptide was completely washed out (Supplementary Fig. 4).

### CAQK binding is specific to sites of brain injury

To further characterize the binding of CAQK to the injured brain area, we carried out overlay binding experiments with silver nanoparticles (AgNPs) conjugated with CAQK (CAQK-nanoparticles (NPs)) on mouse brain sections (Supplementary Fig. 5a–c). CAQK-NPs showed strong binding to the injured brain sections, whereas the binding of control NPs (CGGK-NP) was negligible (Fig. 2f–h). Low binding of CAQK-NPs to brain sections from normal animals was observed, suggesting the presence of low levels of the peptide binding epitope in normal brain, and its elevation upon injury. Similar binding pattern of CAQK-NPs was also observed in the CCI model (Supplementary Fig. 5d,e). Binding specificity was confirmed by inhibiting the CAQK-NP binding with excess of free CAQK, which resulted in near complete inhibition (Supplementary Fig. 5f–h).

### CAQK peptide interacts with components of brain ECM

To identify the potential protein targets of CAQK in the brain tissue, we performed mass spectrometry proteomics analysis of proteins separated from extracts of injured brain by affinity chromatography on immobilized peptides (Supplementary Fig. 6). Table 1 shows a comparison of proteins in eluates from CAQK and control (CGGK) columns. Among the large number of proteins identified, peptides prominent in the CAQK column eluates belonged primarily to the lectican family of chondroitin sulfate proteoglycans (CSPGs^11^). These included versican, associated proteins tenascin-R and the hyaluronan and proteoglycan link protein (Hapln). Versican and Hapln4 were exclusively present in the CAQK column eluates. In normal brain, lectican proteoglycans form extracellular matrix (ECM) complexes known as perineuronal nets (PNN)^12^ around neuronal surfaces and the expression of some of these lectican proteoglycans is upregulated at sites of CNS injury^13^,^14^.

**Figure 3:**
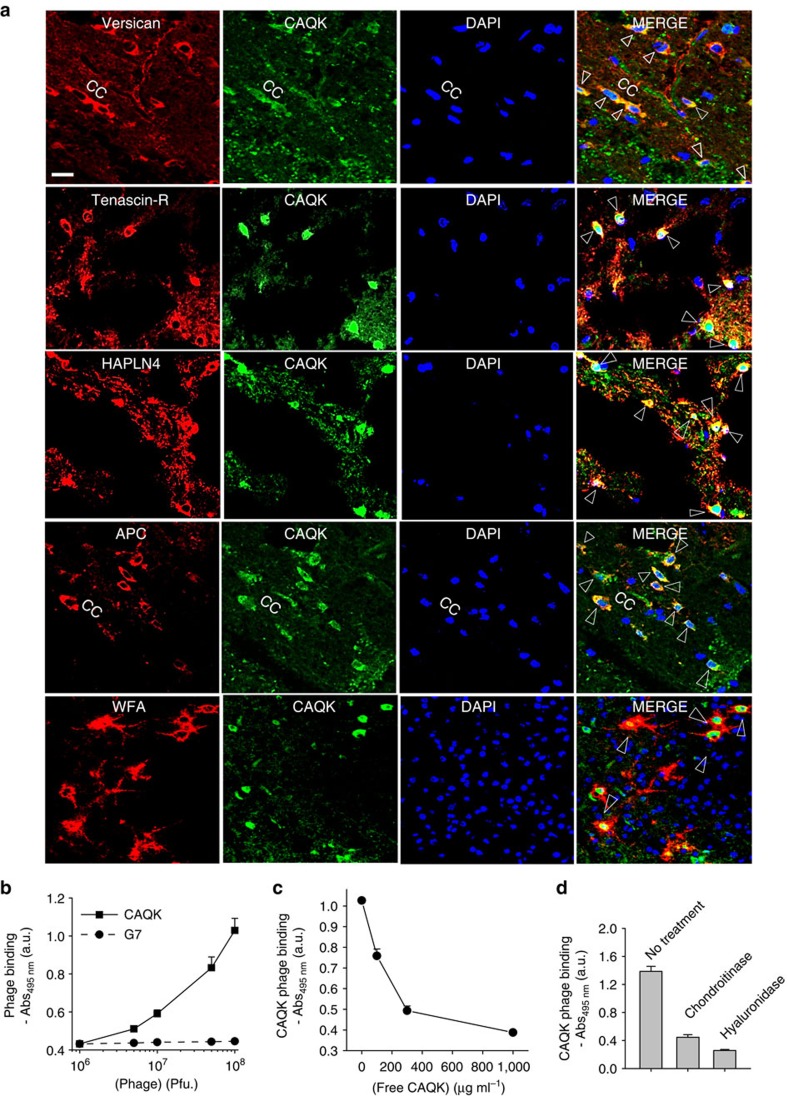
CAQK co-localizes with chondroitin sulfate in PBI. (**a**) Immunofluorescence images of PBI sections showing the corpus callosum (CC) region. The sections were stained for versican, tenascin-R and the hyaluronan proteoglycan link protein, the oligodendrocyte marker (adenomatous polyposis coli (APC)), and Wisteria floribunda agglutinin (WFA; shown in red), FAM-CAQK (green) using anti-fluorescein antibody and counterstained with 4,6-diamidino-2-phenylindole (DAPI; blue). Shown are representative brain sections of injured hemisphere in a PBI mouse injected with FAM-CAQK 6 h after injury. Arrows show co-localization. Scale bar, 20 μm (**b**) Phage binding to ECM formed by U251 cells. The cells were gently dissociated and removed, and the remaining ECM was incubated with phage for 1 h at room temperature, and phage binding was detected following an ELISA protocol. CAQK phage showed higher binding to ECM than control phage. (**c**) Inhibition of CAQK phage binding to ECM by free CAQK peptide. (**d**) Phage binding to ECM is reduced on enzymatic digestion of ECM with chondroitinase ABC or hyaluronidase. Mean±s.e.m. Representative data shown in **b**–**d** is from three experimental repetitions each with three sample replicates.

We confirmed the increase in expression of ECM-associated CSPGs at sites of brain injury by immunostaining. Versican, tenascin-R, and the hyaluronan and proteoglycan link protein (hapln4), all of which are components of the brain ECM complex upregulated following an injury, showed high expression in the injured but not the uninjured hemisphere of the brain (Supplementary Fig. 7). The signal from intravenously injected CAQK co-localized with versican, tenascin-R and Hapln4 (Fig. 3a). The peptide signal also co-stained with Wisteria floribunda agglutinin lectin, a marker for PNNs. At the cellular level, FAM-CAQK prominently accumulated at mature oligodendrocytes identified by expression of the adenomatous polyposis coli marker. In several instances, the CAQK binding pattern followed elongated cells that aligned in the direction of the callosal axons (see CAQK with adenomatous polyposis coli and even better with NG2 in Supplementary Fig. 8). Only a few isolated Olig2 and NG2-positve cells, most likely oligodendrocyte progenitor cells, bound the peptide. No CAQK was detected in or around other glial cell populations, including astrocytes (GFAP+) and microglia (Iba+; Supplementary Fig. 8). Collectively, these data suggest that the binding molecule (receptor) for CAQK peptide is present in the PNN complex that is upregulated in brain injuries.

To explore further the association of CAQK with the PNN complex, *in vitro* binding of CAQK phage to ECM produced by U251 human astrocytoma cells was tested. These cells express high levels of versican and other members of the brain ECM (ref. 15), which suggests that these cells are activated in culture. CAQK phage showed significantly higher binding to the U251 ECM than a control phage (Fig. 3b). In addition to providing further evidence for the ECM binding of CAQK, this result indicates that CAQK recognizes the human target. This is not surprising, as peptides are generally not species-specific in their binding properties^16^. Binding to this ECM was specific as it was inhibited with excess free CAQK peptide. Moreover, enzymatic treatment of the ECM with chondroitinase ABC or hyaluronidase resulted in loss of versican staining (Supplementary Fig. 9) and correspondingly reduced CAQK binding (Fig. 3c,d). This suggests that the epitope for CAQK resides in the complex formed by the CSPGs, hyaluronic acid and associated proteins.

### CAQK as a carrier of diagnostics and therapeutics

The accumulation of the FAM label attached to the CAQK peptide suggested that CAQK is capable of delivering low molecular weight compounds into sites of brain injury. To investigate further the translational potential of the CAQK-targeting approach, we first examined CAQK-mediated delivery to brain injury of NPs as a model of both an imaging agent and a drug carrier. CAQK conjugated, silver NPs (mean diameter—20 nm), administered intravenously, showed significantly greater accumulation in injured brain tissue than control NPs (Supplementary Fig. 10). The localization of CAQK-NPs was in excellent agreement with the localization of the free CAQK peptide (Fig. 2b,e). Thus, CAQK targeted NPs mimic the homing ability of the free peptide.

**Figure 4:**
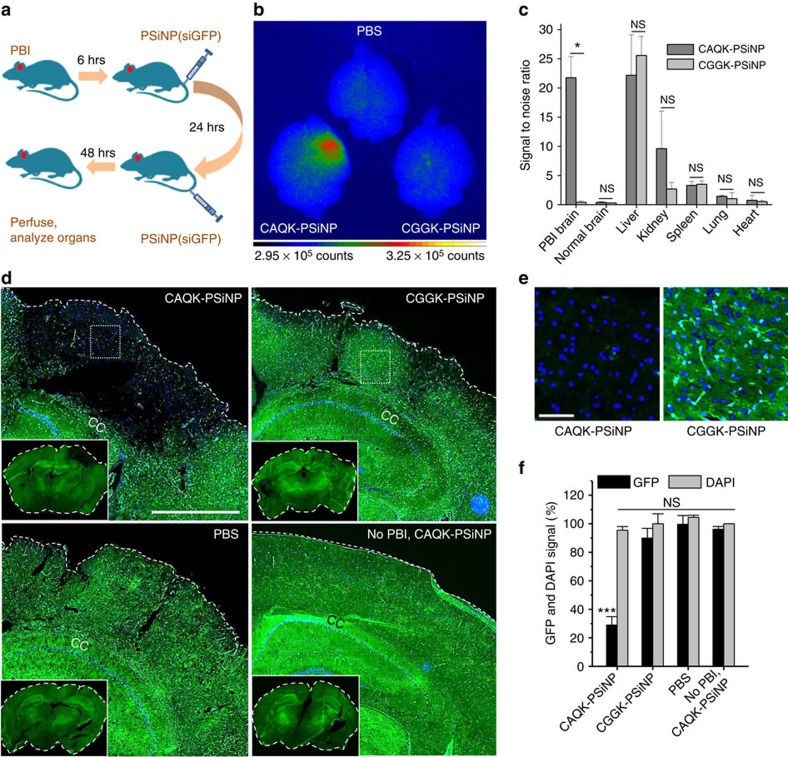
SiRNA delivery in PBI with CAQK-conjugated PSiNPs. (**a**) Schematic of the experimental design for siRNA delivery. (**b**) Gated luminescence imaging of PSiNPs under pulsed laser excitation (*λ*ex=410 nm). PBI mice expressing GFP were injected with vehicle alone (PBS) or PSiNPs conjugated with CAQK (CAQK-PSiNPs) or CGGK (CGGK-PSiNPs). Brains and other organs were isolated 72 h after PBI and analysed for gated luminescence. (**c**) Signal-to-noise ratio (SNR) calculated for the peptide-conjugated PSiNPs in each mouse tissue (relative to PBS control) as described in the Methods section. CAQK-PSiNP showed significantly higher SNR in PBI brains than the control peptide-conjugated group (mean±s.d., *P<0.05, two-tailed Student's *t* test, NS, not significant, *n*=3). (**d**) Microscopic analysis of GFP expression (green) in coronal sections from the animals from panel A counterstained with 4,6-diamidino-2-phenylindole (DAPI; blue). CAQK-PSiNP-injected brains exhibited a large void of GFP in injured area of PBI brain (inset). Scale bar, 500 μm. (**e**) Higher magnification fluorescent images of GFP expression in PBI brain from **d**. Scale bar, 50 μm. (**f**) Mean GFP intensity in injured hemisphere was normalized to corresponding contralateral hemisphere and plotted as percentage GFP expression (*y* axis; ***P<0.001, ANOVA analysis, *n*=3). DAPI (DAPI signal (plotted) showed similar total cell number. Mean±s.e.m., NS, not significant.

To demonstrate the versatility of CAQK system, we tested delivery of oligonucleotides loaded into porous silicon NPs (PSiNPs) as a carrier^17^. Our proof of concept approach was to silence local expression of green fluorescent protein (GFP) systemically expressed in transgenic mice from the CAG promoter^18^. We simulated therapeutic oligonucleotides by using short interfering RNA (siRNA) against GFP loaded in CAQK conjugated PSiNPs (Supplementary Fig. 11a–d). PSiNPs were intravenously injected into the GFP mice with PBI and visualized by time-gated luminescence imaging^19^,^20^ allowing quantification of their accumulation in the excised brains. The imaging showed that CAQK-PSiNPs accumulated in the injuries at markedly higher (35 fold) levels than PSiNPs coated with a control peptide (Fig. 4b). Other tissues, including regions of the brain outside the injury area, showed no significant difference in the accumulation of CAQK and control PSiNPs (Fig. 4c). Confocal microscopy on transverse cortical sections from mice injected with CAQK-PSiNP-siGFP exhibited a large void of GFP expression at the injury site, whereas brains from mice treated with control NPs did not differ from untreated brains (Fig. 4d). We observed 70% silencing of GFP expression by targeting siGFP, whereas minimal silencing was observed with untargeted siGFP and other controls. This silencing was visible across the entire injury and not just in a particular cell type presumably due to the gradual degradation of PSiNP and release of the siGFP over time in the injury. The gene silencing was specific for brain injury, as GFP expression remained unaltered in normal brains or in other major tissues (Supplementary Fig. 11e).

**Figure 5:**
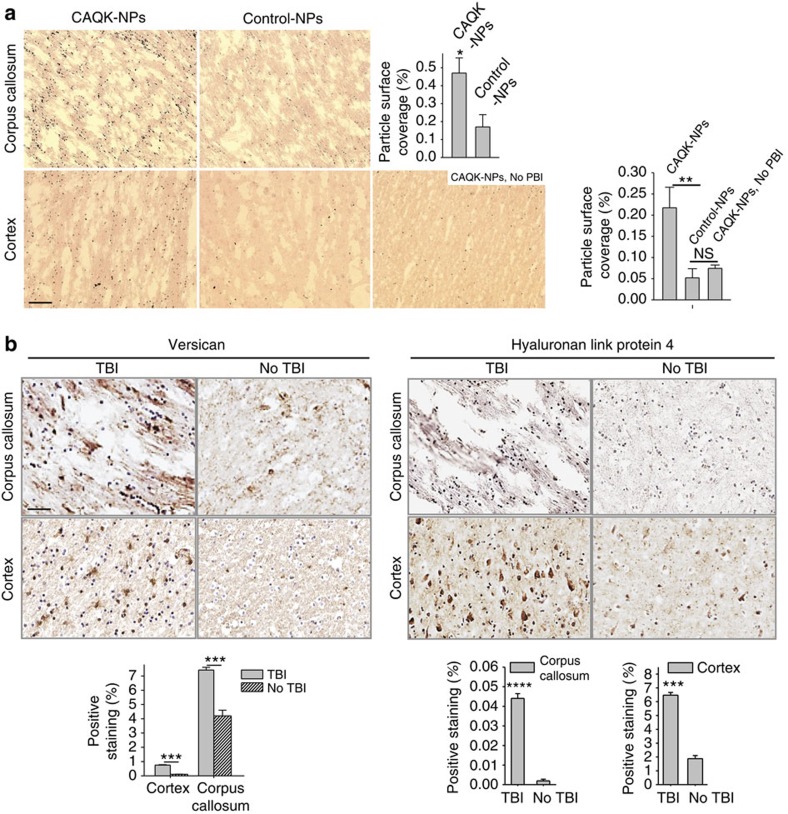
CAQK binds to injured human brains. (**a**) CAQK-conjugated AgNPs (CAQK-NPs) showed higher binding to sections from the corpus callosum and cortex of human brains with TBI than with normal brain or with control NPs. Peptide-conjugated nanoparticles were incubated with formalin-fixed frozen sections from injured and normal brains for *ex vivo* binding. Sections were counterstained with nuclear fast red. The number of particles in each frame were counted and plotted in the bar diagram. Representative photomicrographs are shown. Scale bar, 50 μm (**b**) Versican and hapln4 expression in human brains. Immunohistochemical staining on human brains showed elevated expression of versican and hapln4 in injured brain compared to normal brain tissue. Positive staining was quantified and plotted. Scale bar, 50 μm. Mean±s.e.m., (ANOVA analysis; **P<0.01, ***P<0.001, ****P<0.0001).

Lastly, to examine CAQK recognition of human brain injury, we tested *ex vivo* binding of CAQK-conjugated silver NPs on human cortical sections obtained from a head trauma patient. The CAQK-NPs showed intense binding to the injured brain sections from the cortex and the corpus callosum areas, whereas binding to normal brain sections was minimal (Fig. 5a). Similar to the mouse brains, we observed significant elevation in expression of versican and hapln4 in injured brain than in normal brain by immunohistochemistry (Fig. 5b). These findings confirm CAQK binding to human target and support its potential utility for therapeutic application in humans.

## Discussion

We describe a 4-amino acid peptide that selectively recognizes brain injuries and accumulates at the sites of injury. We show that this peptide, CAQK, enhances the accumulation of systemically administered payloads with chemistries ranging in size from a drug-sized molecule to nanoparticles, and incorporating a variety of imaging and therapeutic functions of potential utility in clinical management of brain injuries. Importantly, the target of this delivery system is expressed both in mouse and human brain injuries.

We used two mouse models in this study. The PBI model mimics gunshot or shrapnel wounds, such as the ones sustained by a warfighter. The blunt cortical impact model more generally reproduces the features of severe TBI. CAQK recognized the injuries in both models, suggesting broad utility across acute brain injuries. The fact that the contralateral hemisphere, unlike normal brain, accumulated some CAQK phage suggests that less severe injuries than the ones we used may also be targeted with CAQK. It remains to be tested whether CAQK also homes to spinal cord injuries, or even demyelinating CNS lesions such as in multiple sclerosis. Importantly, we also show that CAQK recognizes its target in cultured human cells and in cortical sections of injured human brain.

The phage screening in this study revealed a novel aspect of the *in vivo* screening; whereas the previous screens have probed the vasculature of the target tissue, even in the brain^21^,^22^,^23^, the compromised BBB integrity in brain injury allowed the phage to probe the extravascular brain tissue. Moreover, the high-throughput sequencing of the peptide-encoding inserts in the phage genome provided a technical improvement to the screening. One round of selection, as opposed to repeated rounds as previously done, provided a fingerprint of over 200,000 peptide sequences, revealing a striking enrichment of phage displaying the CAQK peptide sequence. Although a cyclic phage library was used, a library of this design contains a minority of linear peptides because stop codons occur within the random insert causing truncation of the cyclic peptide. Additionally, mutations may also change the structure of the peptide. Thus, peptides that do not conform to the general structure of the library are commonly encountered in phage screening^24^,^25^. The recovery of cyclic peptides containing the CAQK motif, in addition to the dominant linear CAQK peptide, suggests that CAQK motif is also active in the context of a cyclic peptide.

BBB disruption is an important contributor to secondary injury following TBI, and therapies to restore BBB functionality are under investigation for neuroprotection^26^. The localized permeability of BBB and the delayed onset of secondary injury provide a window of opportunity for therapeutic intervention. The literature^27^ and our results suggest the duration of the BBB impairment is at least up to 5 days. Within this time window, affinity ligand-based (synaphic) targeting can be an effective drug delivery approach; our results show as high as 35-fold enhancement in the accumulation of systemically administered imaging agents and therapeutics at and around the site of injury.

The concentrating effect of synaphic targeting is likely accounted for by two factors: the peptide can access and bind to its target that allows accumulation of the payload and causes retention at the site of injury. The impairment of the BBB allows all circulating substances to enter the injury area. And, if the peptide receptor is sufficiently abundant relative to the amount of the peptide-drug conjugate used, the binding of the peptide to the receptor can drive payload accumulation beyond what is caused by passive leakage^28^. This is the case in brain injury, where the components of the CSPG complex are overexpressed upon an injury^12^ (Supplementary Fig. 7). The second important factor is the retention effect. As the drug concentration decreases in circulation, the drug is washed out of the injury area^29^. Peptide binding to its target can retain the drug in the injured microenvironment by minimizing this washout (Supplementary Fig. 4). Therefore, the targeting approach in this work encompasses the critical period of healing, which may provide a more lasting therapeutic effect, at least when the therapeutic action is long-acting. Notably, some oligonucleotides, which is one of the types of drugs we successfully delivered in this work, have been shown to remain active for weeks in tissues^30^.

CNS injury results in formation of a CSPG-rich glial scar, which is a major barrier to regeneration^31^. Strategies to prevent the accumulation of CSPGs in injury or dissolve existing deposits have been explored^14^. However, site-specific delivery of the active compounds has been a challenge. The intrinsic affinity of CAQK peptide for CSPG rich areas in injured brain could be effective in directing a CSPG-reducing payload, such as the chondroitinase ABC enzyme^32^. Having successfully targeted nanoparticle payload into brain injuries, we would expect to accomplish the same with proteins, such as chondroitinase. The ability of the present approach to concentrate a payload at the site of brain injury is important for reducing toxicity at off-target sites. An example of an existing therapeutic agent that would benefit from reduced toxicity is the neuroprotective agent brain-derived neurotrophic factor^33^. It has side effects in the normal brain, which CAQK does not target. Thus, our targeting approach has the potential of converting agents with unfavourable pharmacokinetic profile into efficient drugs.

Oligonucleotide-based drugs are a new class of drugs with great potential but hampered by delivery problems *in vivo*. An example is siRNA, a therapeutic modality with the desired characteristics of specificity and potency, but particularly difficult to deliver through systemic circulation. Previous studies on siRNA therapy of brain injuries have either used direct injection into the CNS space or silenced a target present in the brain endothelial cells^34^. The CAQK-mediated targeted delivery of siRNA reported here provides the first evidence of delivery of active siRNA into injured brain tissue from systemic circulation. A number of targets for gene silencing (such as Bcl-2 family proteins, caspases, histone deacetylases (HDAC) and phosphatase and tensin homolog (PTEN)) have been suggested for brain injury^34^. Here, CAQK-mediated siRNA delivery was accomplished by using porous nanoparticles as a carrier. Thus, these results also open up brain injuries for nanomedicine-based therapeutic approaches.

The discovery of CAQK has translational potential because it can direct a payload from systemic circulation to the site of acute brain injury and retain it there for therapeutically relevant timescales. This approach provides an alternative to local delivery, which is invasive and can add complications to the injury. Moreover, the approach could be transferable to human patients, because CAQK recognizes the human target molecule and because the expression of the target appears to be elevated in injured human brain tissues in the same way it is in the mouse injuries.

## Methods

### Brain injury models

All animal experiments were conducted under an approved protocol of the Institutional Animal Care and Use Committee of Sanford Burnham Prebys Medical Discovery Institute. Eight- to ten-week-old male BL6 mice were anaesthetized with 4% isoflurane (Aerrane; Baxter, UK) in 70% N_2_O and 30% O_2_, and positioned in a stereotaxic frame. Using a head restraint, a 5-mm craniotomy was made using a portable drill and a trephine over the right parietotemporal cortex and the bone flap was removed. PBI model was used as described^35^,^36^. Nine needle punctures using a 21G needle were made 3 mm deep according to a 3 × 3 grid, spaced 1 mm in width and 1 mm in height. For TBI, a CCI model was used as described^37^. Mice were subjected to CCI using the benchmark stereotaxic impactor (Impact One; myNeuroLab.com) with the actuator part mounted directly on the stereotaxic instrument. The impactor (3 mm in diameter) tip accelerated down to the 1.0 mm distance, reaching the preset velocity of 3 m s^−1^, and the applied electromagnetic force remained there for the dwell time of 85 ms, and then retracted automatically. The contact sensor indicated the exact point of contact for reproducible results. In both models, facemask anaesthesia (1–2% isoflurane in 70%/30% nitrous oxide/oxygen) was used during the entire procedure and afterwards, the scalp was closed with sutures, anaesthesia discontinued and mice were administered buprenorphine i.p. for pain control. For the first 2 h after injury, mice were closely monitored in their cages.

### *In vivo* phage display

Six hours after brain injury, mice were intravenously injected with 1e10 p.f.u. of a CX7C naïve phage library in 100 μl of PBS. The library was allowed to circulate for 30 min, after which mice were anaesthetized with 2.5% avertin and perfused with PBS intracardially. Brains were extracted, and the tissue surrounding the injury and the corresponding region from the contralateral side was isolated. Tissues were homogenized in LB-NP 40 (1%) and phage was processed as described^7^. Briefly, recovered phages were titered and amplified in *E. coli* BLT5403 and purified for input for second round of screening. The colonies recovered from second round were sequenced using Sanger sequencing (Eton biosciences, San Diego, USA). Alternatively, after first round, the phages in the lysate were rescued by amplification in *E. coli* and peptide-encoding portion of the phage genome was sequenced using Ion Torrent high-throughput sequencing.

### Peptide synthesis and coupling

The peptides were synthesized on a microwave-assisted automated peptide synthesizer (Liberty; CEM, Matthews, NC) following Fmoc/t-Bu (Fmoc:Fluorenyl methoxy carbonyl, t-Bu: tertiary-butyl) strategy on rink amide resin with HBTU (*N,N,N′,N′*-Tetramethyl-*O-*(1*H*-benzotriazol-1-yl)uranium hexafluorophosphate (OR) *O*-(Benzotriazol-1-yl)-*N,N,N′,N′*-tetramethyluronium hexafluorophosphate) activator, collidine activator base and 5% piperazine for deprotection. Fluorescein and biotin tags were incorporated during synthesis at the N-terminus of the sequence. Cleavage using a 95% trifluoro acetic acid followed by purification gave peptides with >90% purity. Peptides were lyophilized and stored at −20 °C.

### Animal experiments for skin and liver injury

For liver injury model, animals were anaesthetized and a midline laparotomy was performed by first cutting the skin, bluntly separating the muscle and then lifting the peritoneum with sterile forceps. A small hole was made into the lifted peritoneum and the hole was carefully expanded (without damage to the internal organs) in both directions along the midline. Once good exposure to liver was obtained, the artery was clamped (Roboz surgical (RS-5420)) and excision wound 2 mm in depth on the surface of one of the livers lobes was made. The clamp was removed from the artery allowing blood to flow. The incisions on peritoneum, muscle and skin were closed. The mice were then placed on a heating pad in their cage and monitored closely until they recovered from anaesthesia.

For skin wounds, induction of anaesthesia was done and the skin was cleaned with alcohol and betadine and four 6- or 8-mm skin biopsies were made to the back skin of the mouse. None of the skin wounds were covered or sutured closed to guarantee optimal and infection-free healing. To test peptide homing, FAM-labelled peptide (50 nmoles) was injected i.v. 6 h after injury and allowed to circulate for 30 min. Mice were perfused intracardially with saline and organs were isolated and analysed by immunostaining.

### Homing studies and tissue sections

Animals were intravenously injected, 6 h after injury, with 50 nmoles of peptide dissolved in PBS, and allowed to circulate for 30 min. Mice were perfused intracardially with saline and organs were isolated and imaged using the Illumatool Bright Light System LT-9900 (Lightools Research). Brains and organs were placed in 4% paraformaldehyde (PFA) at pH 7.4 overnight, washed with PBS and placed in graded sucrose solutions overnight before optimal cutting temperature compound (OCT) embedding. Ten-micrometre-thick sections were cut and analysed by immunofluorescence. For a complete histological analysis, sections were stained with the Movat pentachrome kit (American Mastertech Inc.) following the manufacturers instructions.

### Immunofluorescence

Frozen sections were permeabilized using PBS-Triton (0.2%), blocking was carried out using 5% blocking buffer: 5% bovine serum albumin, 1% goat serum, 1% donkey serum in PBS-T. Primary antibodies were incubated in diluted (1%) blocking buffer overnight at dilutions 1/100 or 1/200 at 4 °C, washed with PBS-T and incubated with secondary antibodies diluted 1/200 or 1/500 in 1% diluted buffer for 1 h at room temperature, subsequently washed with PBS-T, counterstained with 4,6-diamidino-2-phenylindole 1 μg ml^−1^ in PBS for 5 min, washed with PBS, mounted using mounting media (Vector Biolabs) and imaged on a confocal microscope (Zeiss LSM-710). Staining was done using the following antibodies and reagents: Fluorescein (Invitrogen A889), Versican (abcam, ab177480), hapln4 (R&D systems, AF4085), tenascin0-R (R&D systems, AF3865), Wisteria floribunda agglutinin (Sigma, L1516), NG2 and olig2 (gift from Dr William Stallcup at SBPMDI), GFAP (Dako, Z0334) and MBP (Millipore - MAB386).

### CLARITY imaging of brain

CLARITY was performed on freshly extracted brain tissues as described^10^. Briefly, mice were intravenously injected with FAM-labelled peptides 6 h after brain injury. After 30-minute circulation, mice were intracardially perfused with PBS and hydrogel solution (Acrylamide (4%), bis (0.05%), VA-044 Initiator (0.25%), paraformaldehyde 4% in PBS). Following perfusion, the tissues were incubated in the hydrogel solution at 4 °C for 2–3 days. At UCSD Neuroscience Core and Light Microscopy Facility the tissues were then degased with nitrogen and incubated at 37 °C for 3–4 h for polymerization. Samples were then passively cleared in 4% SDS solution for ∼4 weeks until the tissue became transparent. Finally, the samples were washed in PBS-T for 2 days and incubated in gradient glycerol solutions: 30, 50 and 80%, ∼1 day each and stored in 80% glycerol at room temperature until imaged on a confocal microscope (Leica SP5).

### Affinity chromatography and proteomics

For identifying CAQK binding proteins, mouse brains with brain injury were collected 6 h after injury. Using liquid nitrogen, the brains were crushed and ground into powder using a mortar and pestle. Next, brain tissue was lysed in PBS containing 200 mM *n*-octyl-beta-D-glucopyranoside and protease inhibitor cocktail (Roche) as described^38^. The cleared lysate was loaded on to CAQK or control peptide (CGGK) coated Sulfolink-agarose beads (Pierce, Waltham, MA) and incubated at 4 °C for 3–4 h. The column was washed with wash buffer (75 mM octyl-beta-D-glucopyranoside and protease inhibitor cocktail in PBS) followed by washing with 0.5 mM control peptide in wash buffer to remove nonspecifically bound proteins. The bound proteins were eluted with 2 mM-free CAQK peptide. The eluted factions were pooled, their protein concentration determined by using bicinchoninic acid protein assay (Thermo Fischer) and the samples were digested using the filter-aided sample preparation method^39^. Finally, the digested samples were desalted, dried and subjected to liquid chromatography–mass spectrometry (MS)/MS analysis at the Proteomics Core facility of the Sanford Burnham Prebys Medical Discovery Institute. All mass spectra were analysed with MaxQuant software version 1.5.0.25. The MS/MS spectra were searched against the *Mus musculus* Uniprot protein sequence database (version July 2014).

### Phage binding to ECM

Cells grown as confluent monolayer in a 96-well plate were gently removed by a enzyme-free cell dissociation buffer (Thermo Fisher Scientific) and plates blocked with 200 μl of 0.5% bovine serum albumin in PBS with 0.005% T20 for 1 h at 37 °C. Phage was incubated in the plate at 4 °C for overnight and unbound phage was removed by washing three times with 200 μl of PBST. The bound phage was detected by incubating with an in-house generated anti-T7 phage antibody for 1 h at 4 °C. Following washing, horseradish peroxidase-labelled anti-rabbit IgG (Sigma-Aldrich) was diluted 1:1,000 in PBS, and 100 μl was added to the wells, followed by 30 min incubation at room temperature and washing three times. Next, 100 μl of o-phenylenediamine dihydrochloride (OPD) silver and gold substrate (Sigma-Aldrich) was added to the wells and incubated at room temperature until visible colour was observed (<30 min). Adding 50 μl of 1 M H_2_SO_4_ stopped the reaction and the plate was read at 495 nm (FlexStation 3 Reader, Molecular Devices, Sunnyvale, CA, USA). For enzymatic digestion, chondroitinase ABC (2 U ml^−1^, Sigma) or hyaluronidase (500 U ml^−1^) was added to the plate for 3 h at 37 °C. The plate was then washed with PBST three times before incubation with phage.

### Silver nanoparticles synthesis and targeting

AgNPs with PEG coating and peptide functionality were prepared as reported previously with some modifications^40^. AgNPs of ∼20 nm diameter were synthesized by tannic acid reduction of silver nitrate in citrate solution^41^. AgNO_3_ (252 mg) dissolved in 2.5 l water was stirred and heated to 60 °C, then 50 ml water containing tannic acid (6.1 mg) and trisodium citrate dihydrate (1 g) was added. After 3 min, the solution was brought to a boil for 20 min. Final optical density at 400 nm was ∼10. Lipoic PEG amine (51.9 mg, 3,400 g mol^−1^, Nanocs) was reduced in 84 mM tris-carboxylethyl phosphine (TCEP pH 7.0, Sigma) in 4.1 ml water for 3 h. AgNPs were portioned to 500 ml and heated to 50 °C, then lipoic PEG amine solution (0.79 ml) was added, followed by 0.25 ml 0.5 M TCEP. After 30 min, the solution was cooled to room temperature. Tween 20 (T20, 0.25 ml and 10% in water) and 20 ml 2 M NaCl were added, and incubated overnight at 4 °C. AgNPs were concentrated 50-fold and purified by stirred cell (Millipore) with a 100 kDa membrane into 0.5 × PBS with 0.005% T20 and 5 mM TCEP, then passivated with 0.03 mM *N*-acetyl-L-cysteine methyl ester (Sigma), and 0.10 mM tetracysteine peptide (acetyl-CCPGCC-amide, LifeTein), washed at 20 kRCF and resuspended at 300 OD at 405nm in 0.05 M phosphate buffer with 0.005% T20. This product could be stored at least 6 months at 4 °C. A bifunctional linker was reacted with the amine to introduce maleimide groups (NHS-PEG-Mal, 5 kDa JenKem USA), washed by centrifugation, and reacted with cysteine peptide (FAM-x-CAQK-NH2) or a control thiol-containing peptide, or L-cysteine (Sigma). The product was washed in PBS with 0.005% T20 (PBST), filtered (0.22 μm), with a typical final optical density of 150 at the Ag plasmon peak of 400 nm. We estimated this concentration to be ∼30 nM in AgNPs using an extinction coefficient of 5 × 10^9^ M^−1^ cm^−1^ for spherical silver obtained from ref. 42. Fixed tissue sections were stained for Ag using Silver Enhance (Thermo Fischer), counterstained with Nuclear Fast Red (Sigma) and mounted in DPX (Sigma). AgNP signal in tissue sections was quantified by Image J software by isolating grey pixels that represent Ag. For animal experiments, 35 nM of peptide-conjugated AgNPs were injected intravenously in mice 6 h after PBI. The particles were allowed to circulate for 2 h, and the mice were perfused and the brains were isolated. Silver accumulation in the brain was analysed by silver staining autometallography with counterstaining with nuclear fast red (Sigma).

### Tissue section overlay of silver nanoparticles

Overlay experiments to analyse *ex vivo* binding were carried out on frozen brain tissue sections following the same protocol for immunofluorescence staining described above taking the nanoparticles as if they were the primary antibody and using no secondary antibody. Peptide-coated AgNPs in PBS-T at a 1nM concentration were used and the incubation time was 1 h at 37 °C. Sections were imaged with fluorescent microscopy by looking at intrinsic emission from the FAM tag on the peptide.

### Synthesis and functionalization of porous silicon nanoparticles PSiNPs

Single-crystalline highly doped *p*-type silicon wafers (∼1 mΩ cm resistivity, <100> polished, boron-doped) were purchased from Virginia Semiconductor, Inc. PSiNPs were prepared by electrochemical perforation etching of the silicon wafers, as described previously^43^. Briefly, the silicon wafer was anodically etched in an electrolyte consisting of 3:1 (by volume) of 48% aqueous HF:ethanol. Etching was carried out in a Teflon etch cell that exposed the polished silicon wafer surface, using a platinum coil counter electrode. The silicon wafer was contacted on the backside with a strip of aluminium foil. The etching waveform consisted of a square wave in which a lower current density of 50 mA cm^−2^ was applied for 1.8 s, followed by a higher current density of 400 mA cm^−2^ for 0.36 s. This waveform was repeated for 140 cycles, generating a perforated porous silicon film with alternating layers of high and low porosity. The resulting porous nanostructure was removed from the silicon substrate by applying a current density of 3.7 mA cm^−2^ for 250 s in an electrolyte consisting of 1:30 (by volume) of 48% aqueous HF:ethanol. The free-standing porous silicon films were then fractured to the desired size (nominally 150 nm) by ultrasonication, and the resulting nanoparticles were oxidized by immersion in aqueous borax solution to activate photoluminescence^44^.

### Peptide conjugation and siGFP loading to PSiNPs

An aliquot of PSiNPs (2 mg ml^−1^ in ethanol) was mixed with 20 μl of 3-(ethoxydimethyl)-propylamine silane by vortex overnight at room temperature. The amine-terminated nanoparticles were rinsed three times with ethanol and then further reacted with 1 ml of succinimidyl carboxy methyl ester-polyethylene glycol-maleimide (10 mg ml^−1^ in ethanol) for 2 h, followed by rinsing with ethanol and deionized water (three times each). An aqueous solution of the peptides (500 μl, 1 mg ml^−1^, either CAQK or control) was added to the resulting nanoparticles and mixed by vortexing for 2 h to conjugate the peptide via the free cysteine residue at the terminal group of the peptide^45^. Peptide conjugation was confirmed by measuring fluorescence of a FAM tagged to the peptide. The amount of peptide on nanoparticles was estimated to be ∼70 nmol mg^−1^ (peptide/PSiNPs). The oligonucleotide siGFP was electrostatically loaded to the positively charged porous inner structure of the nanoparticles by mixing with siRNA solution (200 μM) at 4 °C for 24 h. The loading amount of siGFP was∼7 wt% (siGFP/PSiNP), which was determined by measuring absorbance at 260 nm. Note that the PEG linkers were attached only onto the outer surface (not the inside pores) of PSiNPs due to their large molecular weight and long-chain length relative to the pore size (∼12 nm). 3-(ethoxydimethyl)-propylamine silane and succinimidyl carboxy methyl ester-polyethylene glycol-maleimide (SCM-PEG-Mal, MW 5000) were purchased from Sigma-Aldrich and Laysan Bio, respectively, and used as received without further purification. RNase-free water was purchased from Thermo Fischer (Carlsbad, CA). Small interfering RNA against GFP (siGFP) was purchased from Dharmacon. The sequences for the sense and antisense strands of siGFP are: 5′-GGCUACGUCCAGGAGCGCACCdTdT-3′ (sense) and 5′- UGCGCUCCUGGACGUAGCCTTdTdT-3′ (antisense).

### Characterization of PSiNPs

Transmission electron microscope images were obtained on JEOL-1200 EX II operating at 120 kV. Dynamic light scattering (DLS, Zetasizer ZS 90, Malvern Instruments) was used to determine the hydrodynamic size and Zeta potential of the nanoparticles. Photoluminescence and fluorescence spectra were obtained using a QE pro spectrometer (Ocean Optics). Concentration of siRNA was determined by measuring absorbance at 260 nm using a spectrometer (NanoDrop 2000, Thermo Fisher Scientific) based on the OD_260_ standard curve of siRNA.

### *In vivo* siRNA targeting and analysis

Transgenic CAG-GFP mice were purchased from The Jackson Laboratory (stock #006567). Brain injuries were done as described above. Peptide-conjugated and siRNA-loaded PSiNPs (300 μg) were administered twice via tail-vein injections at 6 and 24 h post injury (*n*=3). Three days after injury, mice were perfused, organs harvested and fixed for downstream analysis. The tissues were imaged under a time-gated imaging set-up and the GFP expression was analysed by researchers blinded to the experimental groups.

### Gated luminescence imaging of silicon nanoparticles (GLISiN)

Gated luminescence images were acquired from a custom-built time-domain imaging system using an intensified CCD camera (iSTAR 334 T, Andor Technology Ltd.), as reported^19^. A tunable laser consisting of a tripled Nd:YAG-pumped optical parametric oscillator (Opolette 355, Opotek Inc.) were used as an excitation source at a repetition rate of 10 Hz, which was synchronized and triggered with the CCD. The Andor SOLIS software was used to control time delays and acquisition conditions and to analyse signal-to-noise ratio. Mouse tissues were placed on a black polystyrene plate, and bright field (under ambient light) and gated luminescence (under excitation with pulsed laser, *λ*_ex_=410 nm) images were taken.

### Human tissue experiments

Formalin-fixed human brain tissues were obtained from the Brain Tissue Repository maintained by the Center for Neuroscience & Regenerative Medicine (CNRM) at the Uniformed Services University of the Health Sciences (USU) in Bethesda, MD. The TBI case is from a patient with moderate TBI (automobile accident) who died at age 72. The control case is from a 63-year-old male without any neurologic diagnosis or any signs of TBI on detailed neuropathologic evaluation. Fixed tissues were cryopreserved and sectioned for overlay binding with AgNPs as described above. For immunohistochemistry, an antigen retrieval step was done before incubation with anti-hapln4 and anti-versican antibodies.

### Statistical analysis

All data represents mean value±s.e.m. All the significance analysis was done using Statistica 8.0 software, using one-way ANOVA or two-tailed heteroscedastic Student's *t* test. The details of the statistical tests carried out are indicated in respective figure legends.

### Data availability

The data that support the findings of this study are available from the corresponding author on request.

## Additional information

**How to cite this article:** Mann, A. P. *et al.* A peptide for targeted, systemic delivery of imaging and therapeutic compounds into acute brain injuries. *Nat. Commun.* 7:11980 doi: 10.1038/ncomms11980 (2016).

## Supplementary Material

Supplementary Information Supplementary Figures 1-11

## Figures and Tables

**Table 1:** CAQK binds to brain ECM proteins.

**Protein name**	**UniProt ID**	**Gene name**	**Log**_**2**_ **LFQ intensity**	
			**CGGK column**	**CAQK column**
Versican core protein	Q62059	VCAN	—	25.98
Hyaluronan and proteoglycan link protein 4	Q80WM4	HAPLN4	—	19.84
Tenascin-R	Q8BYI9	TNR	24.14	26.71

Proteins belonging to the PNN complex were identified by peptide-affinity chromatography and mass spectrometry analysis on mice PBI brains. The label free quantification (LFQ) intensities were derived using MAXQuant software and averaged for three technical replicates. Intensities are plotted on log_2_ scale. Empty column denotes protein was not detected.
