# Supplementary material for: A peptide for targeted, systemic delivery of imaging and therapeutic compounds into acute brain injuries
Source: Nat Commun. 2016 Jun 28;7:11980. doi: 10.1038/ncomms11980 (PMC4931241; doi:10.1038/ncomms11980)
Supplement: Supplementary Information — Supplementary Figures 1-11 [file ncomms11980-s1.pdf]

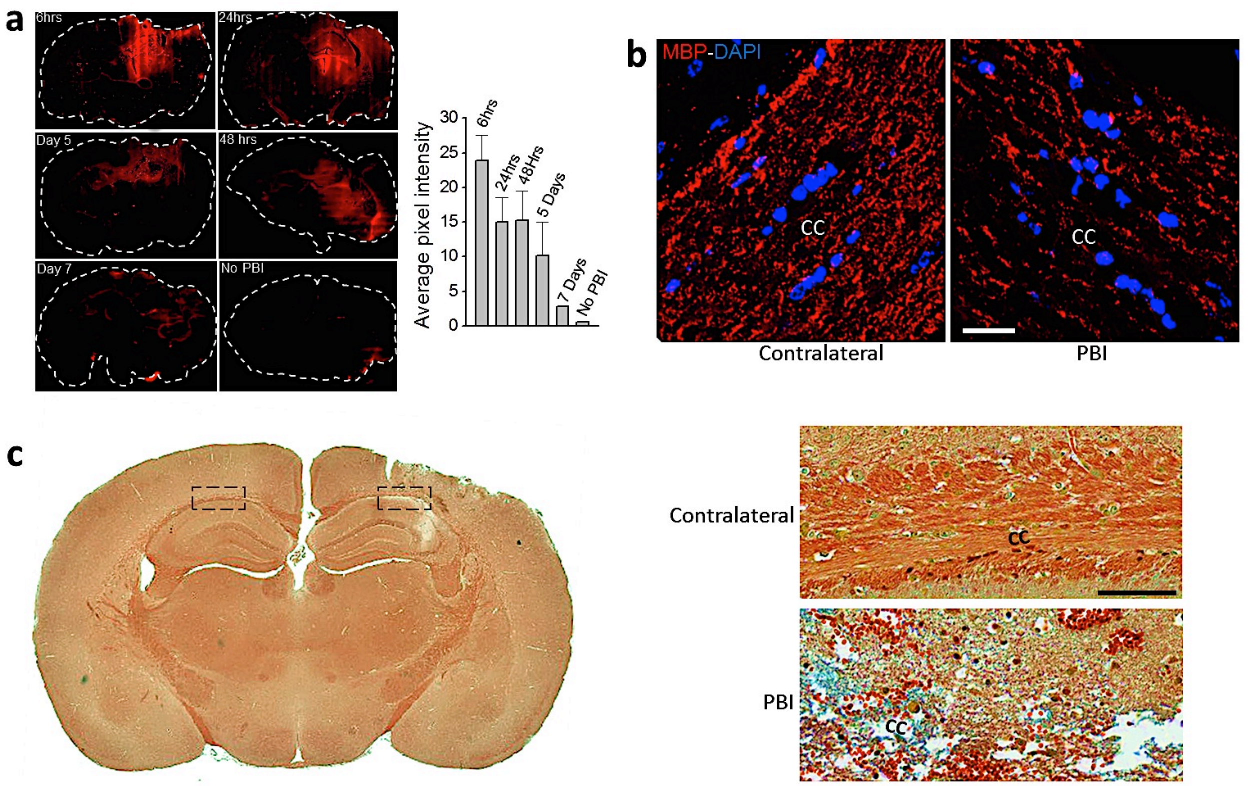

Supplementary Figure 1. Characterization of penetrating brain injury (PBI) (a) Blood brain barrier leakage in PBI. Brains isolated from mice after different time points after PBI stained for mouse IgG (red). Shown are representative images of entire brain. Total fluorescence intensity was quantified and plotted. (Mean ± SEM, *n*=3) **(b)** Demyelination occurs in *corpus callosum* in injury. Immunofluorescence of the injured brain showing the *corpus callosum* (CC) region in the injury side (right panel) and the contralateral side (left panel), six hours after injury. Sections was stained for myelin basic protein (MBP, shown in red) and counterstained with DAPI (blue). Scale bar – 20 µm **(c)** Histology of a perfused brain six hours after PBI, showing injured and contralateral side. Higher magnification in (right panel) shows the corpus callosum (cc) region from contralateral side (top) and PBI brain (bottom) from C. Sections were stained with Movat Pentachrome kit (black: elastic fibers and nuclei, yellow: collagen, blue: mucins, red: muscle, intense red: fibrinoid). Blue staining represents alcian blue, which stains for acidic polysaccharides such as chondroitin sulfate and hyaluronic acid, which are overexpressed in PBI. Scale bar - 100 µm.

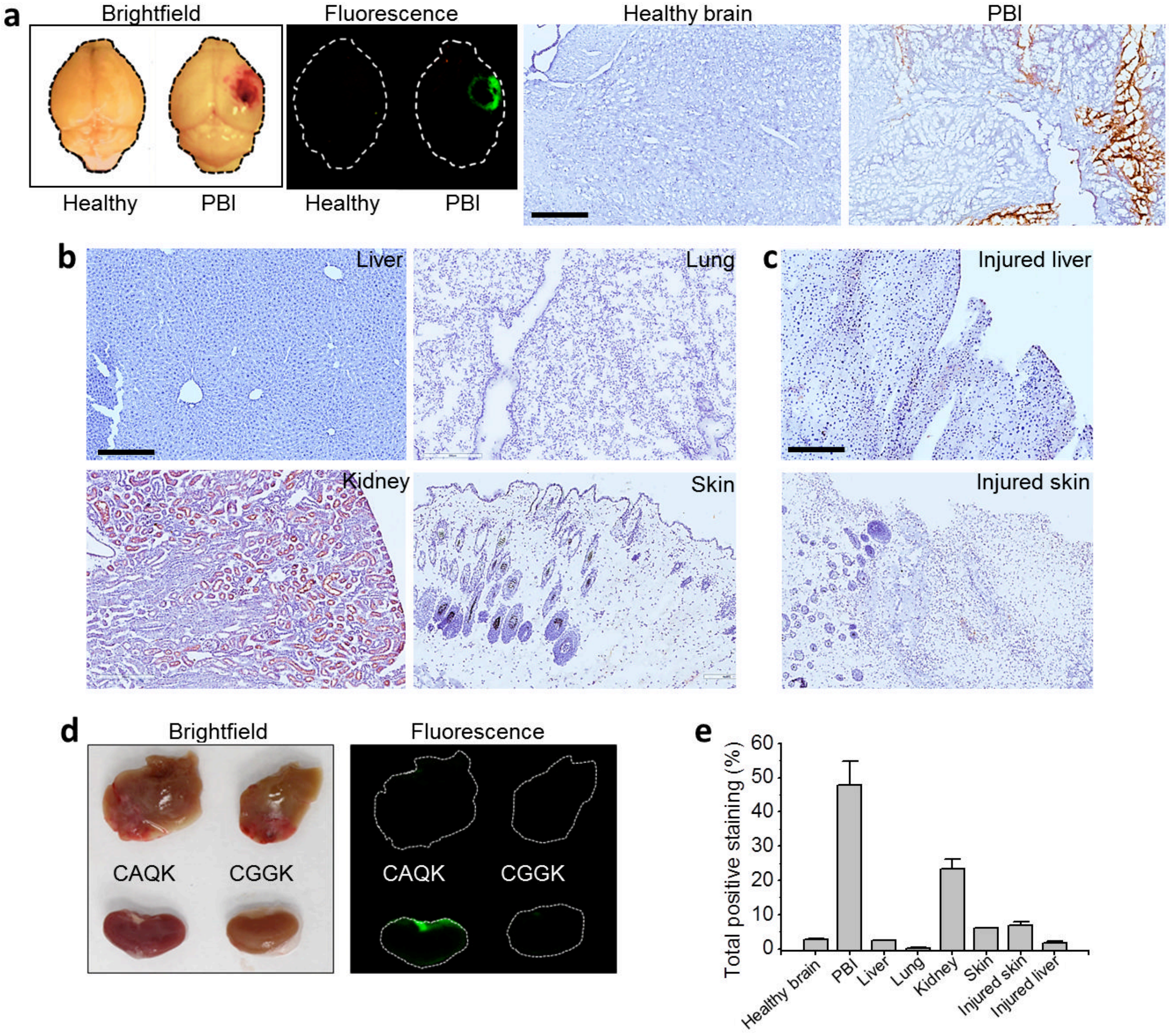

Supplementary Figure 2. CAQK accumulation in brain and other tissues. FAM-labeled peptides were injected in PBI and non-PBI mice 6 hours after injury and allowed to circulate for 30 minutes. Mice were perfused, and the brains were excised and imaged using an Illumatool Bright Light System in the green channel **(a)**. Cryosections from brain and other organs **(b)** were analyzed for accumulation of peptide by immunohistochemical staining for FAM (brown). **(d)** FAM-labeled peptides were injected in mice with liver injury 6 hours after injury and allowed to circulate for 30 minutes. Mice were perfused, and the brains were excised and imaged in the green channel. All sections counterstained with hematoxylin (blue). Scale bar – 300 µm. Signal from immunohistochemical staining was quantified and plotted in **(e).**

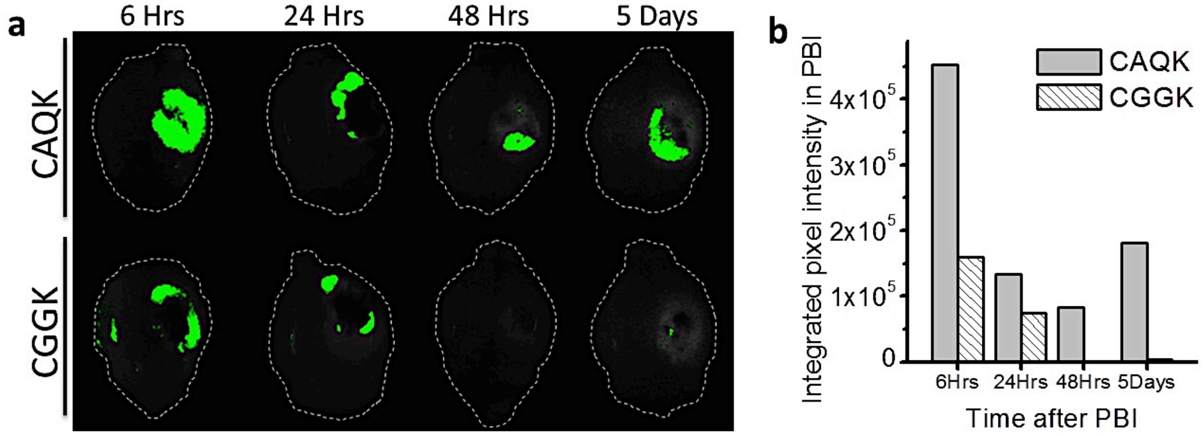

Supplementary Figure 3. Time course of CAQK accumulation in PBI. FAM-CAQK peptide and a control peptide FAM-CGGK were injected at different time points after PBI and allowed to circulate for 30 minutes. Mice were perfused, and the brains were excised and imaged using an Illumatool Bright Light System in the green channel (**a**). Signal intensity was quantified by taking the integrated pixel intensity in the injury in the green channel of the images (**b**).

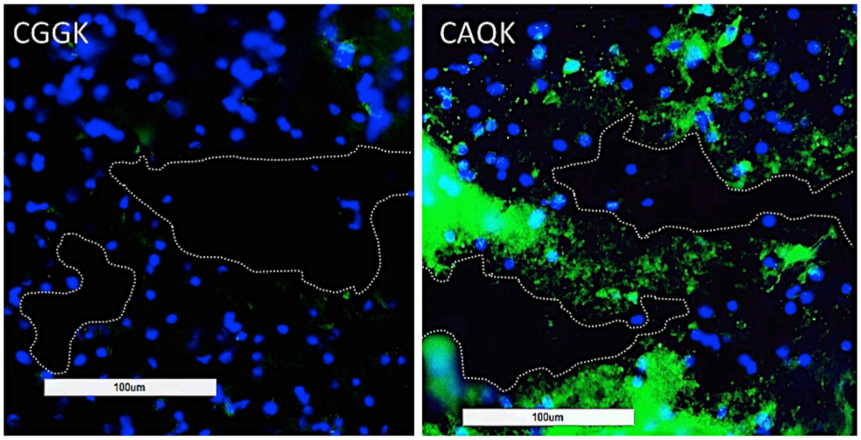

Supplementary Figure 4. CAQK retention in brain injury. FAM-CAQK peptide and control peptide FAM-CGGK were injected 6 hours after PBI and allowed to circulate for 180 minutes. Brains were excised without perfusion and imaged for peptide signal (Green channel, anti-FITC). Shown are the injured hemispheres, in the regions around corpus callosum.

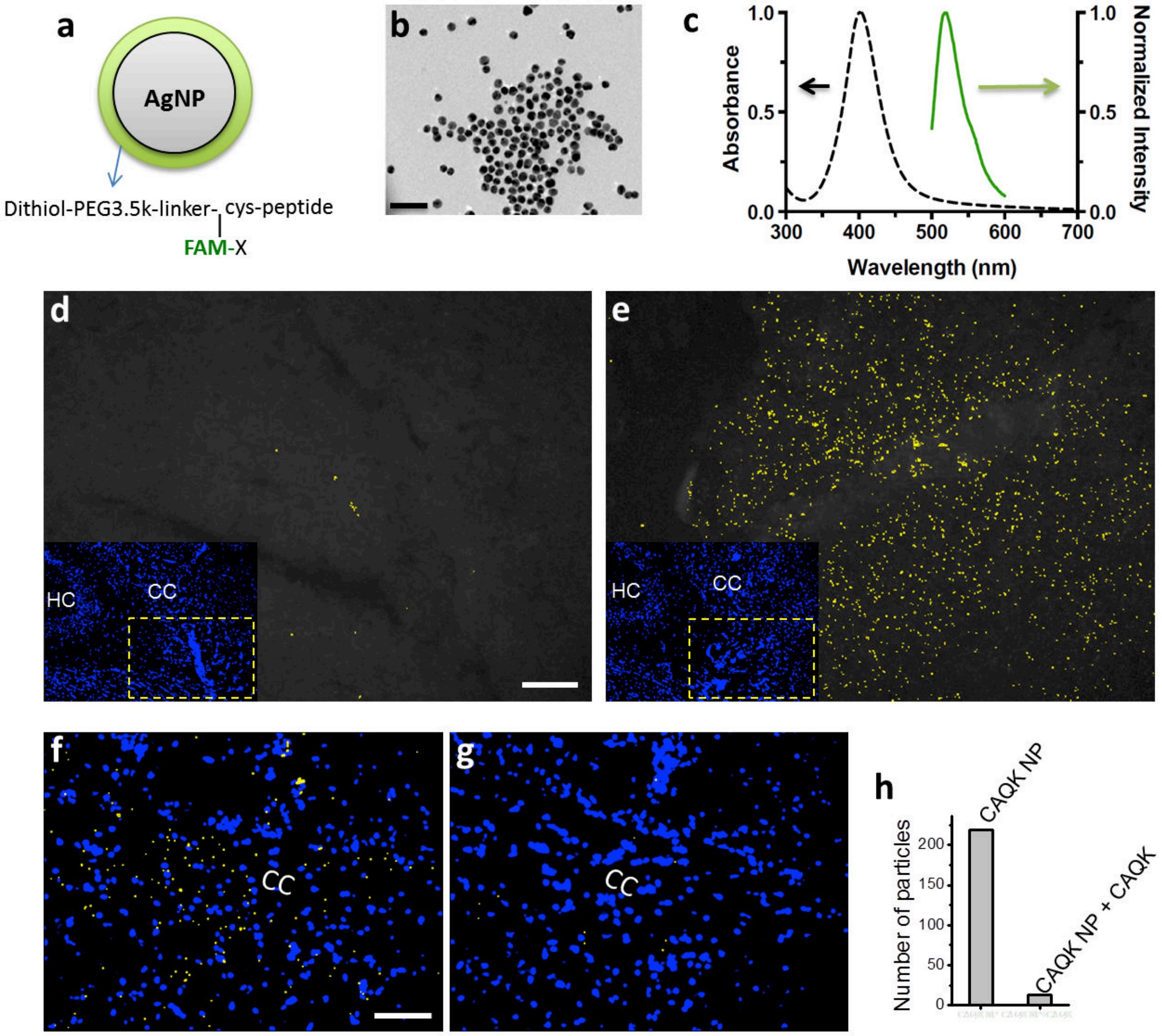

Supplementary Figure 5. Characterization and binding of CAQK conjugated AgNPs. (a-c) Characterization of peptide-conjugated AgNPs. **(a)** Schematic representation of the AgNP platform used, consisting of Ag core functionalized with dithiol-PEG3.5k-Cys-FAM-peptide. **(b)** Transmission electron microscopy image of the AgNPs dried on a *Formvar* coated copper grid. **(c)** Absorbance and emission spectra of the functionalized AgNPs, showing the plasmon resonance peak (black dotted curve) characteristic of spherical AgNPs of this size and the emission peak (green solid curve) in the green region of the spectrum (520 nm), attributed to fluorescein from the FAM-peptide. Overlay binding experiments using silver nanoparticles (NP) conjugated with control peptide (CGGK-NPs) **(d)** and CAQK peptide (CAQK-NPs) **(e)** on fresh frozen contiguous brain sections from CCI injury. FAM-peptide-conjugated NPs (20 nM final concentration) were incubated, for 30 minutes on frozen sections. Slides were thoroughly rinsed with PBS and counterstained with DAPI (blue; the DAPI channel is shown separately in the inset). Green-emitting NPs were pseudo colored to yellow for higher color contrast and the color threshold was enhanced using Image J in the same way for all samples. **(f-h)** CAQK-NP binding to PBI sections is specific. Panel f

and g show CAQK-NP binding on contiguous PBI sections before **(f)** and after **(g)** addition of a 10-fold excess (1.5 µM) of free unlabeled CAQK. Scale bar – 50 µm. CC: *Corpus callosum*, HC: Hippocampus

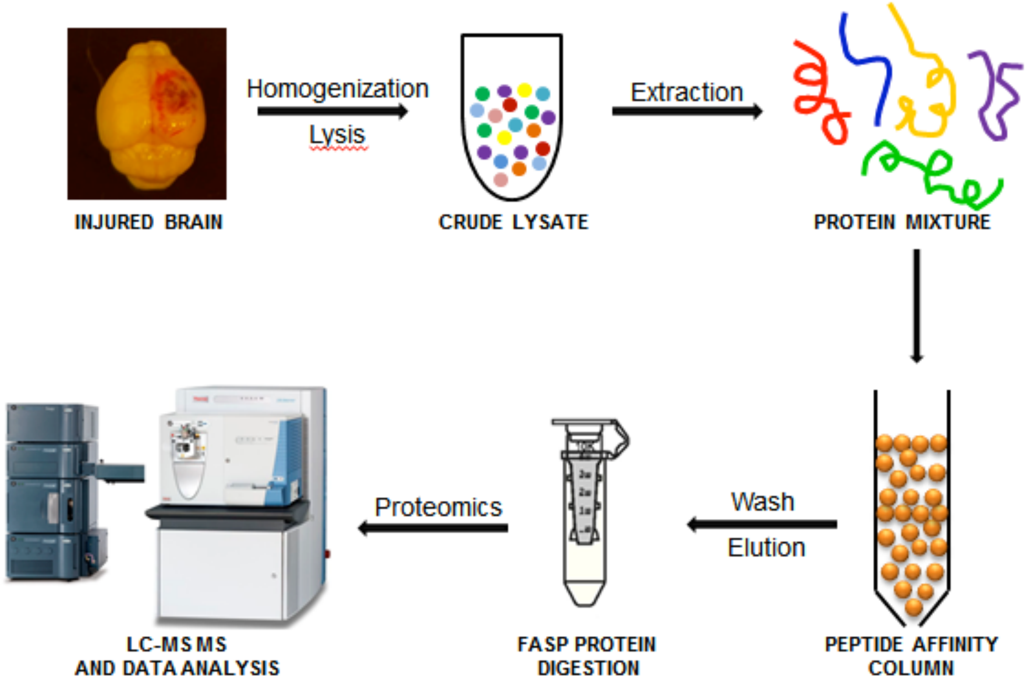

Supplementary Figure 6. Schematic for identification of CAQK receptor from a PBI brain. See methods section for details.

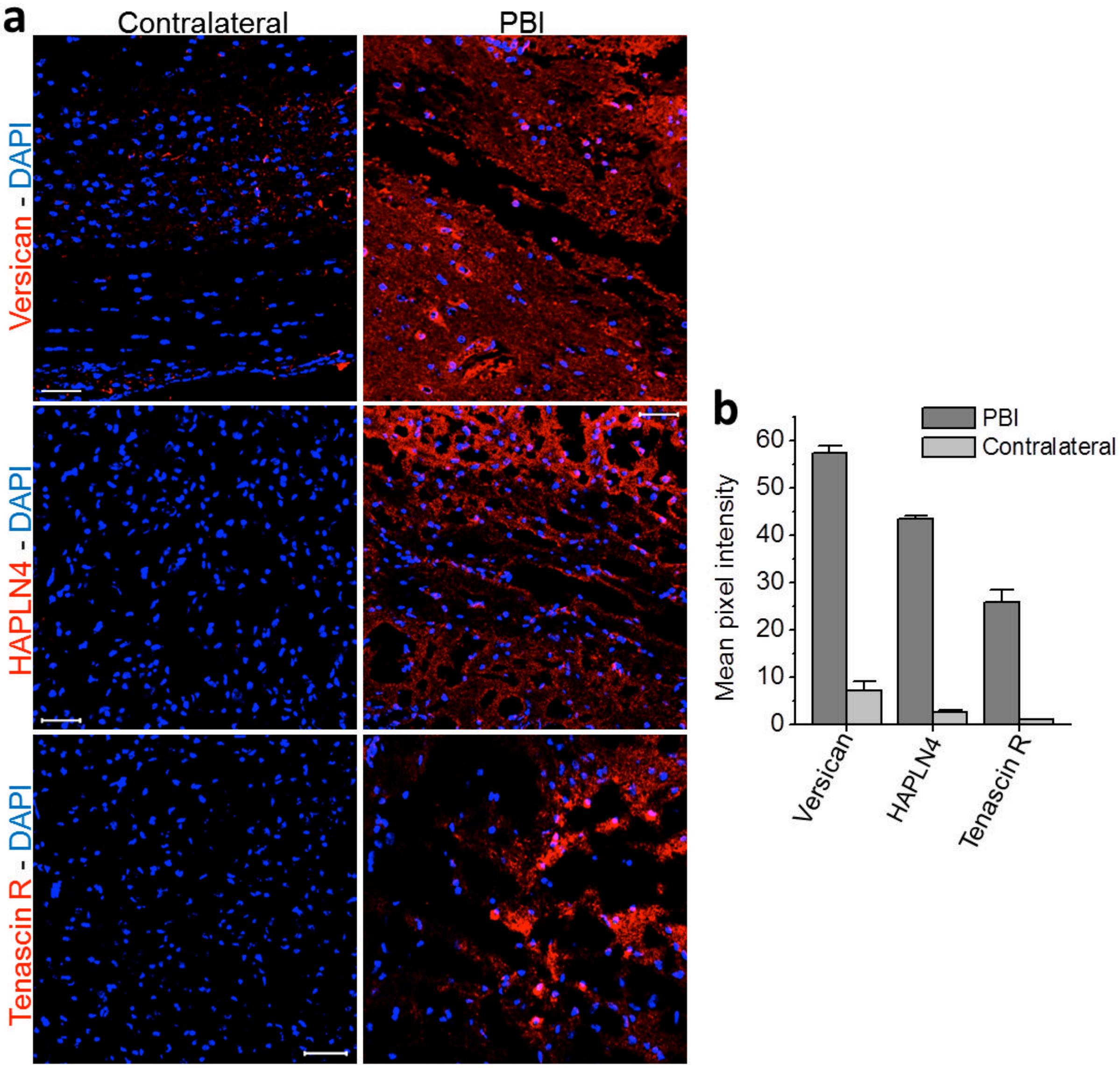

Supplementary Figure 7. Protein expression of PNN components in PBI brain. Shown are representative immunofluorescence images of versican, hapln4 and tenascin R expression (in red) in contralateral and injured hemisphere in PBI brain. Frozen sections from perfused mice brains six hours after PBI were immunostained and analyzed by confocal microscopy. Scale bar – 50 µm. Signal intensity was quantified by taking the integrated pixel intensity in the red channel of three images **(b).** Mean ± SEM.

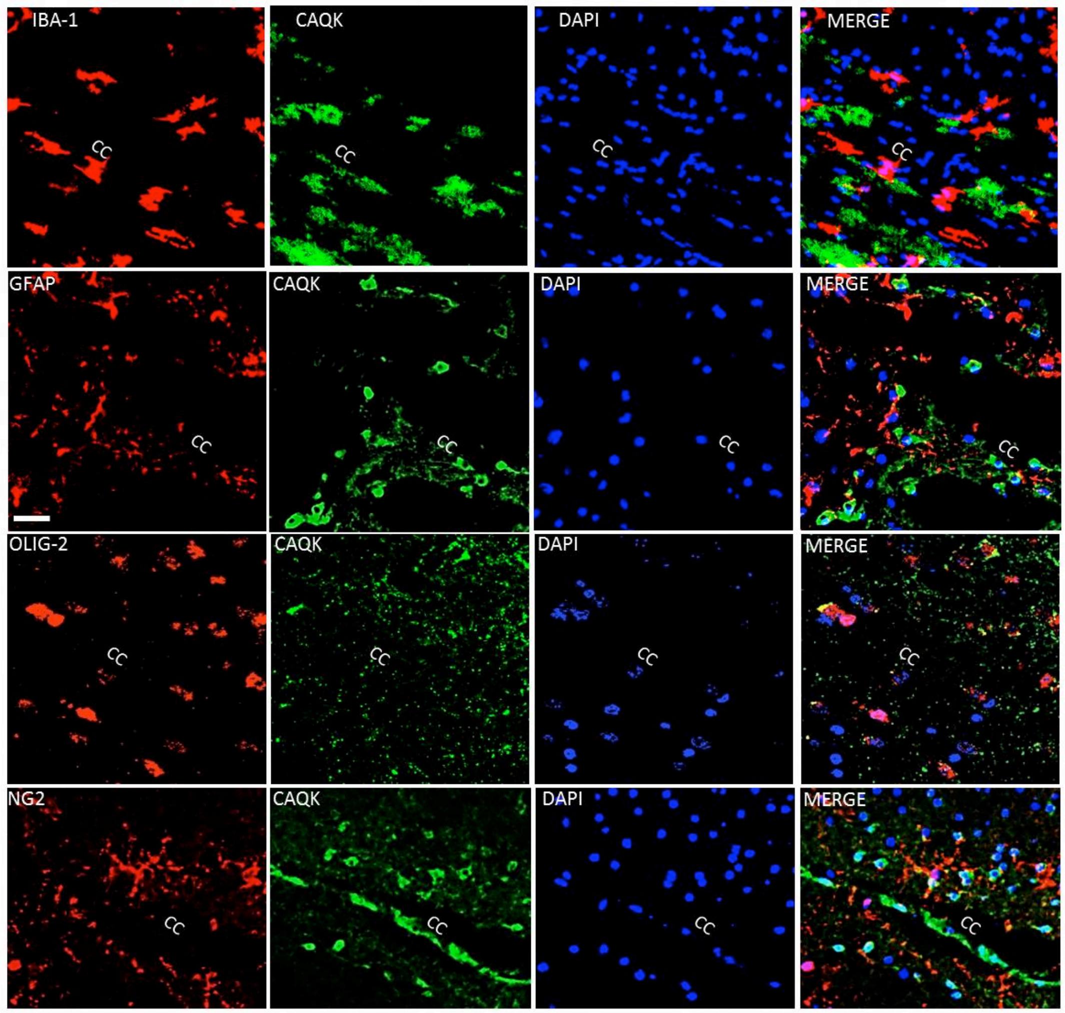

Supplementary Figure 8. CAQK does not co-localize with non-oligodendrocyte glial cells in PBI. Immunofluorescence of PBI brain showing the *corpus callosum* (CC) region in the injured hemisphere six hours after injury. Frozen sections from PBI mice injected with FAM-CAQK were stained for Iba-1, GFAP, Olig-2 and NG2 (shown in red), and for FAM-CAQK (green) using anti-fluorescein antibody and counterstained with DAPI (blue). Scale bar – 20 μm.

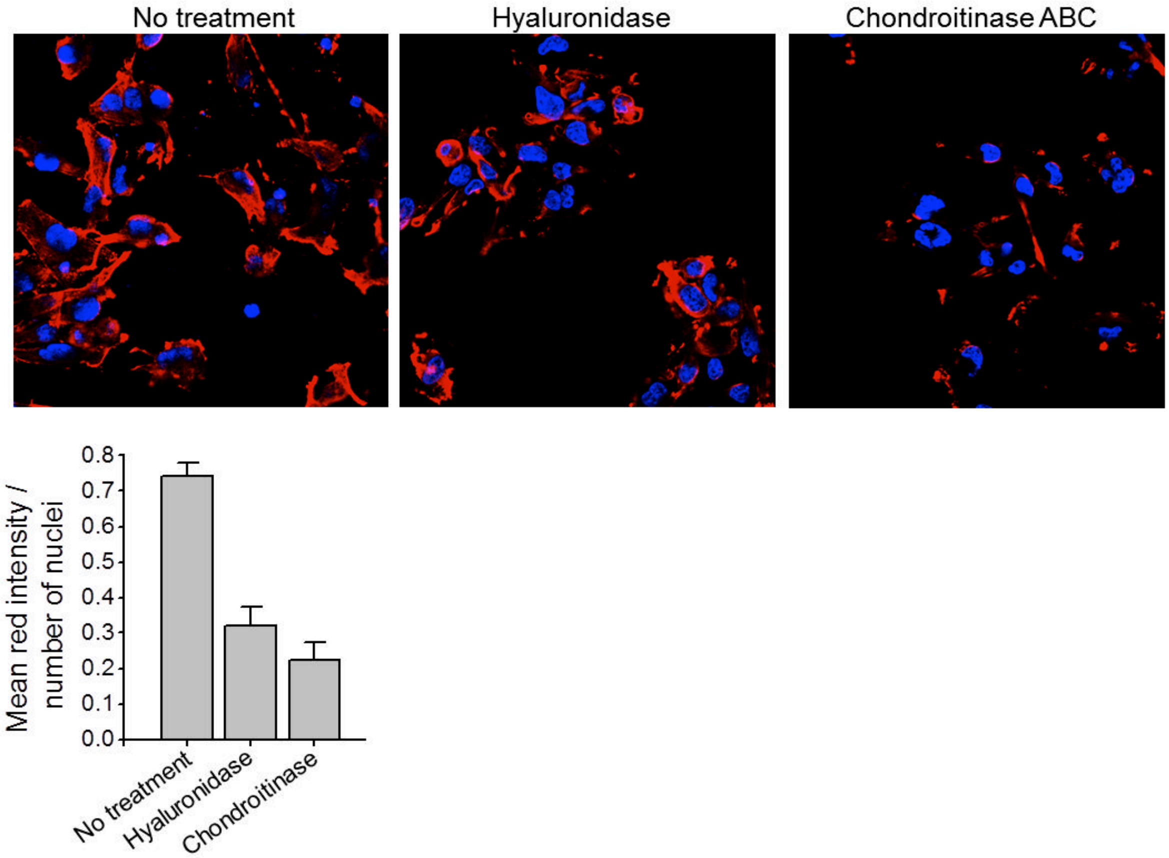

Supplementary Figure 9. Versican expression in U251 cells. U251 cells grown as confluent monolayer were stained for versican expression and analyzed by immunofluorescence after no treatment or treatment with hyaluronidase (50U/ml) or chondroitinase ABC (3U/ml) for 1 hour at 37°C. Versican (red) was quantified and plotted in the bar graph. Cells were counterstained with DAPI. Representative images shown from three experiments.

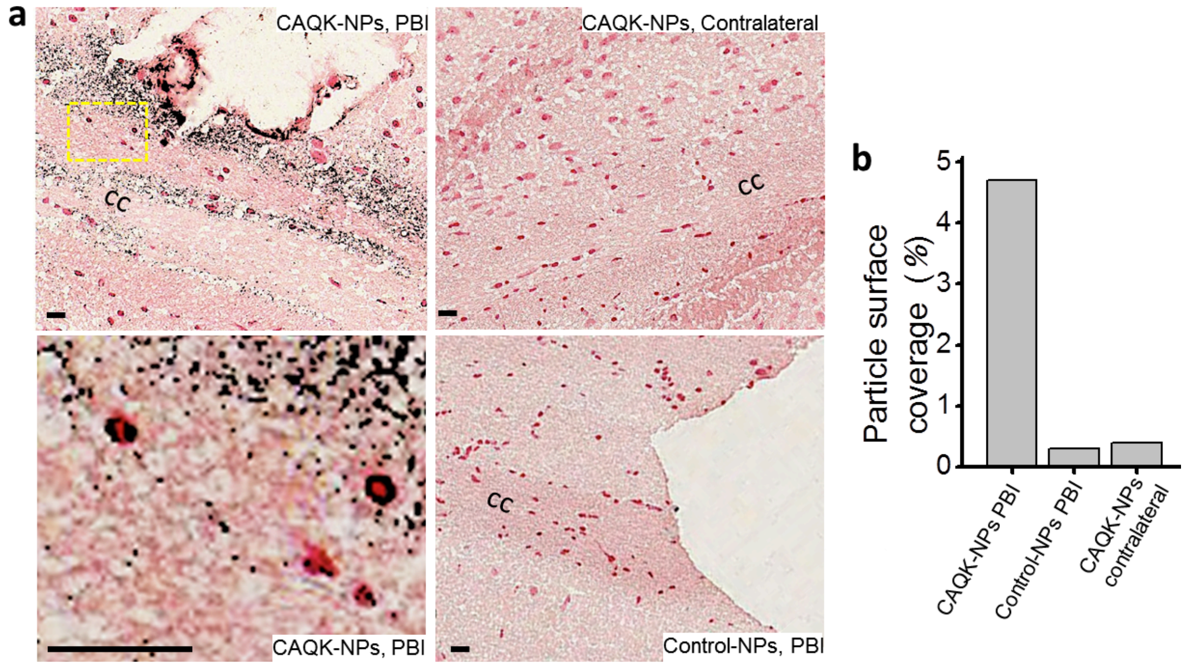

Supplementary Figure 10. CAQK mediated delivery of nanoparticles to PBI. (a-b) Silver nanoparticle delivery to PBI. Silver nanoparticles conjugated with CAQK (CAQK-NPs) or a control peptide (control-NPs) were injected i.v. 6 hours after PBI and allowed to circulate for two hours before perfusion (n=3). In the brain sections shown, silver signal was amplified by autometallography and the sections were counterstained with nuclear fast red. **(a)** Shown are representative sections with CAQK-NPs in the injured hemisphere around the *corpus callosum* (CC; top left panel; high magnification, bottom left panel). The controls include CAQK in the contralateral hemisphere (top right panel) and control-peptide NPs in the injured hemisphere (bottom right panel). The CAQK-NPs appear as round, dark spheres of approximately 300nm, attributed to initial silver seeds from injected nanoparticles. Scale bar – 20 µm. **(b)** Percentage of surface covered by nanoparticles in each frame was quantified using Image J.

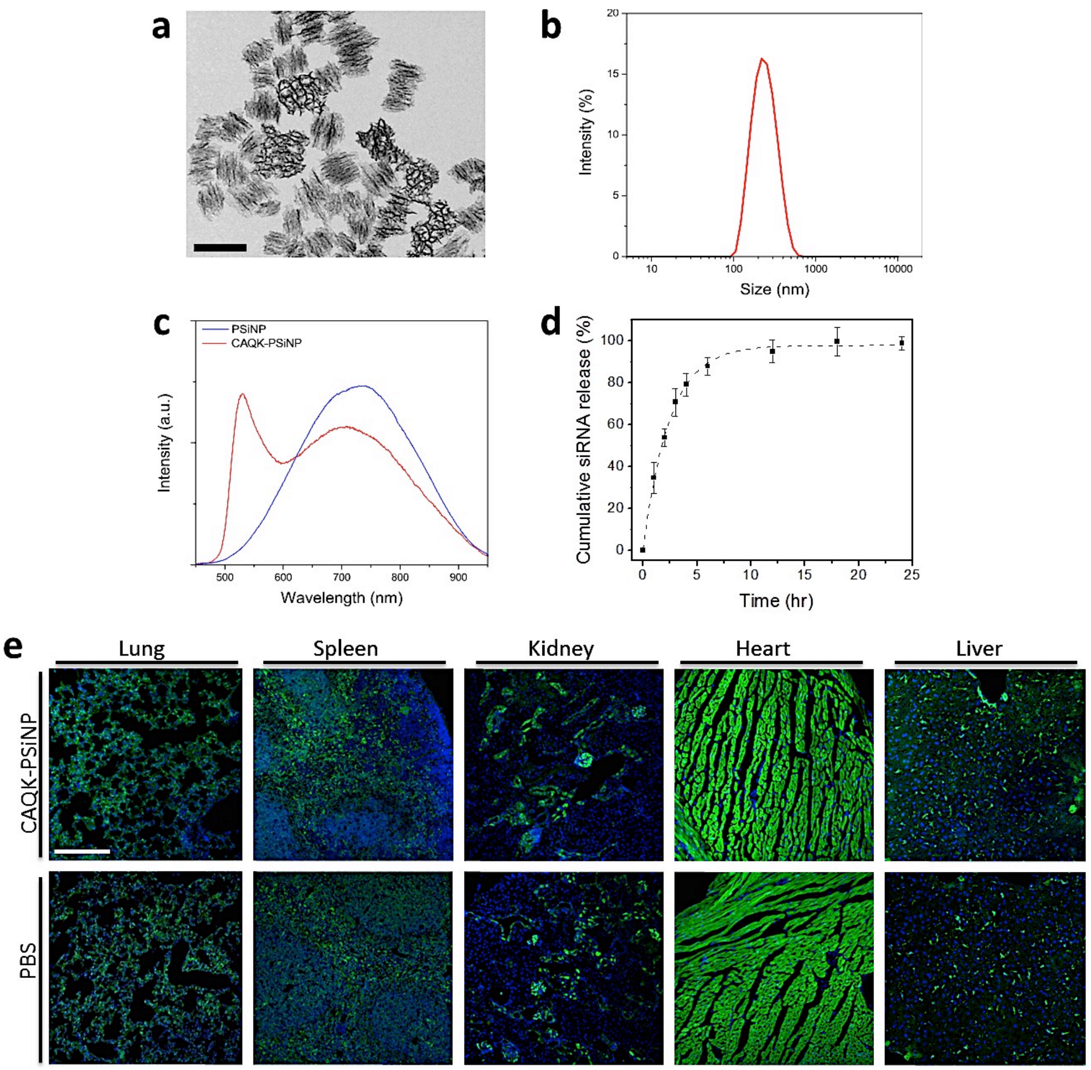

Supplementary Figure 11. PSiNP characterization and PSiNP-mediated siRNA delivery to tissues other than the brain. (a-c) Characterization of CAQK peptide-conjugated porous silicon nanoparticles (CAQK-PSiNPs) using transmission electron microscopy **(a)**, size distribution measured by dynamic light scattering **(b)** and photoluminescence spectra (λ_{ex} = 365 nm) of PSiNP and CAQK-PSiNP **(c)**. FAM label on the peptide appeared in the emission spectrum at ~ 530 nm and was included to allow estimation of conjugation efficiency to PSiNP, as described in methods section. **(d)** Release of siRNA from PSiNPs was analyzed by Dy677-labeled siRNA loaded into PSiNPs and incubated in PBS at 37°C. The released siRNA was obtained from supernatant at different time points. **(e)** GFP expression in major tissues after CAQK-PSiNP-mediated siRNA delivery. Representative fluorescence images showing GFP expression in the organs of CAQK-PSiNP treated mice as compared to untreated mice. Frozen sections were counterstained with DAPI and analyzed for GFP expression under confocal microscopy. Scale bar – 200 µm.
